# Editorial: Well-being and education: current indications and emerging perspectives

**DOI:** 10.3389/fpsyg.2024.1496914

**Published:** 2024-10-02

**Authors:** Eirini Karakasidou, Michael Galanakis, George Tsitsas

**Affiliations:** ^1^Department Psychology, Panteion University, Athens, Greece; ^2^Department of Social Sciences, Neapolis University of Paphos, Paphos, Cyprus; ^3^Department of Economy and Sustainable Development, Harokopio University, Kallithea, Greece

**Keywords:** well-being, resilience, school settings, positive education, counseling psychology in education

## Introduction

In today's rapidly changing world, schools are more than just places of education; they are critical environments where students develop intellectually, emotionally, and socially. The COVID-19 pandemic has underscored the importance of psychological support in schools, highlighting the urgent need for practices that bolster wellbeing and mental resilience among both students and educators. As educational institutions cope with the difficulties of the pandemic and rising inflation, which exacerbate stress and uncertainty, the need for novel approaches to mental health and wellbeing in schools has never been greater (Viner et al., 2020).

Positive psychology, dedicated to understanding what makes life most worthwhile, provides many techniques and strategies that are especially well-suited for use in education. This research, which promotes strengths, resilience, and wellbeing, has the potential to transform schools into caring settings in which both children and teachers may thrive (Seligman et al., 2009).

## Positive psychology: cultivating resilience and wellbeing in education

Positive Psychology offers a variety of tools and methods that can be effectively integrated into the school environment to enhance wellbeing and resilience. The principles of Positive Education, a branch of Positive Psychology focused on applying its concepts in educational settings, hold great promise for transforming schools (Waters, 2011).

One of the key strengths of Positive Psychology is its focus on character strengths, positive communication, and self-compassion, all of which can be nurtured in students and teachers alike (Park and Peterson, 2006; Neff and Germer, 2013). By fostering these qualities, schools can create a more supportive and resilient environment, enabling all members of the school community to cope better with the challenges they face (White and Waters, 2015).

## New tools and methods for enhancing wellbeing

To meet the current challenges, there is a growing need to develop and implement new tools, practices, and methods to enhance the wellbeing and psychological resilience of students, teachers, and parents. Research and proposals from the international scientific community are crucial in this endeavor (Jennings and Greenberg, 2009; Kim and Asbury, 2020; Lee, 2020). Key areas of focus in enhancing wellbeing and resilience in educational settings include the prevention of teacher burnout by developing interventions that help educators manage stress, avoid burnout, and maintain their passion for teaching (Skaalvik and Skaalvik, 2017). Additionally, integrating Positive Psychology and stress management techniques into the school curriculum can assist both students and teachers in coping with daily pressures (Shankland and Rosset, 2017). Team interventions for psychological resilience are also essential, fostering a sense of community and support among students. Encouraging the identification and use of character strengths in schools can build self-esteem and create a positive school culture for both students and teachers. Positive communication strategies are crucial for enhancing relationships and creating a more supportive environment while teaching compassion and self-compassion can improve mental health and cultivate a nurturing school culture. Lastly, applying Positive Psychology tools to build parental resilience and wellbeing ensures that parents can support their children effectively, contributing to the overall health of the educational ecosystem (Parent et al., 2016).

## Building a positive organizational culture in schools

Beyond individual interventions, there is a need to cultivate a positive organizational culture within educational institutions (Waters, 2015). This involves creating an environment where wellbeing is prioritized, and students, teachers, and parents all feel valued and supported. By fostering a culture of positivity, schools can become bastions of mental health and resilience, helping their communities navigate the challenges of today's world (Seligman et al., 2009).

## Overview of studies

This editorial will explore 16 studies focusing on various aspects of wellbeing, stress, resilience, and mental health in students and teachers. Topics include burnout prevention and art therapy, as well as the cultural values shaping student self-efficacy. These studies offer fresh perspectives on the interventions and frameworks that can be used to improve education. Several studies focus on students' mental health and stress experiences, with a particular emphasis on how these can be managed or mitigated through specific interventions.

Gong and Geertshuis explored the unique stressors students studying abroad face. Offshore international students often experience both distress and eustress (positive stress), and this study emphasized the importance of understanding and differentiating these experiences. Similarly, Jones et al. delved into the psychological challenges faced by third-culture kids. It identified resilience and family functioning as key mediators in coping with stress and adjustment issues, providing valuable insights into how family dynamics can support mental wellbeing.

Beltrán-Ruiz et al. underlined the importance of attachment and compassionate approaches, suggesting that attachment-based therapies may significantly reduce psychological distress in university students. They offer a promising approach to enhancing student wellbeing through a focus on compassion and emotional connection.

Academic burnout is a recurring theme in the studies included in this Research Topic. Kushida and Troster shed light on the alarming rates of burnout among medical students who face unique stressors due to the intensity of their academic programs—highlighting the prevalence of burnout in this population. Additionally, Kourea et al. proposed the BENDiT-EU program as a preventive tool to reduce burnout and promote wellbeing among students and staff in their study. This program provides a framework for addressing burnout at both individual and institutional levels.

The wellbeing of teachers is equally crucial in maintaining a healthy educational environment. Li presented a comprehensive analysis of how self-efficacy and resilience prevent burnout among teachers. By emphasizing emotion regulation strategies, this study underscores the importance of supporting teachers' mental health to improve their wellbeing and professional performance. Furthermore, Zhang and Luo explores resilience in teaching, offering valuable insights into how educators can cultivate resilience to buffer the effects of occupational stress.

Several studies explore innovative interventions designed to improve wellbeing in educational settings. Mo and Ko explores the use of art therapy as a tool for emotional expression and stress relief. The paper highlights how creative outlets like mandala art therapy can foster emotional regulation and offer students a calming, reflective practice.

In a similarly novel approach, Chute et al. demonstrate how animal-assisted interventions can reduce momentary stress and enhance emotional wellbeing among students. This study suggests that animals may provide a comforting presence that helps students manage immediate stress, promoting relaxation and reducing anxiety.

Cultural values also play a critical role in shaping educational experiences. Jin et al. take a global perspective, examining how cultural values influence student self-efficacy. This study's findings suggest that culturally informed interventions may be necessary to optimize self-efficacy and academic success across diverse student populations.

The COVID-19 pandemic has profoundly affected education worldwide, creating new challenges for students and teachers. Pöysä et al. compares how educators in two countries experienced stress and support during the pandemic. The study underscores the importance of institutional support in mitigating teacher stress during times of crisis.

Students have also faced significant challenges due to the pandemic. Wang et al. explores how resilience and loneliness moderated students' emotional experiences during the lockdown. The findings emphasize the importance of fostering resilience and addressing loneliness to support students' mental health during such unprecedented times.

The studies in this Research Topic highlight several critical themes for promoting wellbeing, managing stress, and building resilience in educational settings. First, innovative interventions, such as art therapy, animal-assisted programs, and compassion-based therapies, hold significant promise for enhancing mental health. Second, fostering resilience in students and teachers is essential for coping with stress and preventing burnout, particularly in the face of ongoing challenges like the COVID-19 pandemic.

Cultural considerations also play an essential role in shaping how students and educators experience and manage stress. As the study on cultural values and self-efficacy demonstrates, interventions must be tailored to the cultural contexts of the individuals they aim to support. Finally, institutional support for teachers and students alike is crucial. The pandemic has shown that during times of crisis, the level of support provided by educational institutions can significantly impact the mental health and wellbeing of their communities.

The diverse range of topics covered in these 16 studies reflects the growing interest in exploring the mental health challenges students, and educators face in today's educational environments. By understanding the causes of stress and burnout and identifying effective interventions, academic institutions can create healthier, more supportive environments where students and teachers can thrive. These papers provide a robust foundation for further research and action to enhance wellbeing, manage stress, and foster educational resilience.

## Conclusion

Positive Psychology offers a promising path forward for schools seeking to enhance the wellbeing and resilience of their communities. Integrating new tools, methods, and practices into the school environment can create a more supportive, resilient, and positive educational experience. This research initiative aims to bring together the latest work in Positive Education, sharing and synthesizing knowledge to benefit educational institutions worldwide. Through this collaborative effort, schools may not only be places of learning but also environments where students, teachers, and parents can thrive amidst the challenges they face.

The education sector has long been a fertile ground for research on wellbeing, stress, and resilience. In recent years, the challenges faced by students and educators have intensified due to numerous factors, including rising academic demands, mental health pressures, and unprecedented disruptions such as the COVID-19 pandemic. The studies presented in this Research Topic provide valuable insights, highlighting the importance of interventions that foster resilience, manage stress, and promote wellbeing in educational settings.

## References

[B1] Jennings P. A. Greenberg M. T. (2009). The prosocial classroom: teacher social and emotional competence in relation to student and classroom outcomes. Rev. Educ. Res. 79, 491–525. 10.3102/003465430832569338293548

[B2] Kim L. E. Asbury K. (2020). ‘Like a rug had been pulled from under you': the impact of COVID-19 on teachers in England during the first six weeks of the UK lockdown. Br. J. Educ. Psychol. 90, 1062–1083. 10.1111/bjep.12381 32975830 PMC7537096

[B3] Lee J. (2020). Mental health effects of school closures during COVID-19. Lancet Child Adolesc. Health 4:421. 10.1016/S2352-4642(20)30109-7 32302537 PMC7156240

[B4] Neff K. D. Germer C. K. (2013). A pilot study and randomized controlled trial of the mindful self-compassion program. J. Clin. Psychol. 69, 28–44. 10.1002/jclp.21923 23070875

[B5] Parent J. McKee L. G. Rough J. N. Forehand R. (2016). The association of parent mindfulness with parenting and youth psychopathology across three developmental stages. J. Abnormal Child Psychol. 44, 191–202. 10.1007/s10802-015-9978-x 25633828 PMC4520790

[B6] Park N. Peterson C. (2006). Character strengths and happiness among young children: content analysis of parental descriptions. J. Happ. Stud. 7, 323–341. 10.1007/s10902-005-3648-6

[B7] Seligman M. E. P. Ernst R. M. Gillham J. Reivich K. Linkins M. (2009). Positive education: positive psychology and classroom interventions. Oxf. Rev. Educ. 35, 293–311. 10.1080/03054980902934563

[B8] Shankland R. Rosset E. (2017). Review of brief school-based positive psychological interventions: a taster for teachers and educators. Educ. Psychol. Rev. 29, 363–392. 10.1007/s10648-016-9357-3

[B9] Skaalvik E. M. Skaalvik S. (2017). Motivated for teaching? Associations with school goal structure, teacher self-efficacy, job satisfaction, and emotional exhaustion. Teach. Teach. Educ. 67, 152–160. 10.1016/j.tate.2017.06.006

[B10] Viner R. M. Russell S. J. Croker H. Packer J. Ward J. Stansfield C.. (2020). School closure and management practices during coronavirus outbreaks including COVID-19: a rapid systematic review. Lancet Child Adolesc. Health 4, 397–404. 10.1016/S2352-4642(20)30095-X 32272089 PMC7270629

[B11] Waters L. (2011). A review of school-based positive psychology interventions. Aust. Educ. Dev. Psychol. 28, 75–90. 10.1375/aedp.28.2.75

[B12] Waters L. (2015). A review of school-based positive psychology interventions. Aust. Educ. Dev. Psychol. 32, 1–19. 10.1375/aedp.28.2.75

[B13] White M. A. Waters L. E. (2015). A case study of ‘The Good School:' examples of the use of Peterson's strengths-based approach with students. J. Posit. Psychol. 10, 69–76. 10.1080/17439760.2014.920408 25431614 PMC4241650

